# Detection of poststroke oropharyngeal dysphagia with swallowing screening by ultrasonography

**DOI:** 10.1371/journal.pone.0248770

**Published:** 2021-03-17

**Authors:** Takao Matsuo, Miwa Matsuyama

**Affiliations:** 1 Faculty of Allied Health Sciences, Division of Speech-Language-Hearing Therapy, Department of Rehabilitation Sciences, Kansai University of Welfare Sciences, Asahigaoka, Kashiwara city, Osaka, Japan; 2 Department of Oral Health Care and Rehabilitation, Institute of Biomedical Sciences, Tokushima University Graduate School, Kuramoto-cho, Tokushima city, Tokushima, Japan; Sapienza University of Rome, ITALY

## Abstract

There are currently no standard evaluation tools for poststroke neurogenic oropharyngeal dysphagia. We previously suggested calculating the relative movements of the hyoid bone and larynx by ultrasonography to evaluate swallowing movement. Swallowing movement is altered in neurogenic oropharyngeal dysphagia. Therefore, the present study aimed to verify whether an ultrasonographic evaluation of swallowing movement facilitates the detection of neurogenic oropharyngeal dysphagia. Eighteen healthy male elderly participants (the healthy group) and 18 male stroke patients diagnosed with neurogenic oropharyngeal dysphagia (the dysphagia group) were enrolled. Participants swallowed 5 mL of liquid and water with an adjusted viscosity and the movements of the hyoid bone and larynx were visualized by ultrasonography. The results obtained revealed significant differences in laryngeal duration (static phase), laryngeal displacement (elevation phase), and the hyoid bone–laryngeal motion ratio (HL motion ratio) between the two groups. A multiple regression analysis was performed to adjust for confounding factors, and laryngeal duration (static phase) and the HL motion ratios were identified as factors affecting dysphagia. In the receiver operation characteristic curve of the two variations, the area under the curve for laryngeal duration (static phase) was 0.744 and the cut-off was 0.26 sec with 72.2% sensitivity and 88.9% specificity; the area under the curve for the HL motion ratio was 0.951 and the cut-off was 0.56 with 88.9% sensitivity and 88.9% specificity. Therefore, the objective evaluation of hyoid bone and larynx movements during swallowing by ultrasonography facilitated the detection of neurogenic oropharyngeal dysphagia.

## Introduction

Poststroke dysphagia is a high risk factor for pneumonia, nutritional imbalance, and dehydration [1–3], and has a poor prognosis and high mortality rate [4,5]. An early swallowing evaluation after hospitalization may reduce the risk of pneumonia [6,7]. In addition to poststroke dysphagia, older patients also present with sarcopenic dysphagia due to reduced muscle mass [8–10]. Therefore, the effective and efficient management of dysphagia is important [11].

Neurogenic oropharyngeal dysphagia (NOD) is the most common poststroke dysphagia, affecting more than 50% of patients in the acute phase of stroke and more than 33% in the recovery phase [12,13].

The accurate diagnosis of NOD is crucial and generally achieved by screening, clinical signs, and device measurements. However, there are currently no standardized NOD evaluation tools [14]. The conventional gold standard swallowing evaluation includes bedside swallowing screening followed by a video fluoroscopic (VF) or videoendoscopic test when dysphagia is suspected [15,16]. Swallowing movement is altered in patients with NOD. For example, laryngeal vestibular closure in the swallowing process may be delayed or lower food transport from the oral cavity to the esophagus. Since VF visualizes movements in the oral cavity and pharynx, it may be applied to the evaluation of NOD. In a meta-analysis conducted by Martino et al. [1], poststroke dysphagia was detected in 37–45% of cases by screening, 51–55% by clinical signs, and 64–78% by devices. Also, it is believed that clinical signs are not effective for swallowing screening in aspiration of suspected severe poststroke NOD cases [17]. Therefore, the inspection of images is vital for the accurate evaluation of swallowing. VF helps to detect movements in the oral cavity and pharynx, but is limited by its specific setting, cost, radiation exposure, and complexity [18].

We previously suggested calculating the relative movements of the hyoid bone and larynx by ultrasonography (US) to evaluate swallowing movement [19]. Our study showed that movement, but not aspiration, was measurable by US, which is very safe, simple, portable, and applicable to bedside measurements [20]. Therefore, it may be used in the screening of dysphagia that allows image inspection for high-quality management.

The present study aimed to verify whether US evaluations of swallowing movement facilitate the detection of NOD. We previously suggested measuring the displacement of the hyoid bone and larynx during swallowing to obtain a “hyoid bone–laryngeal motion ratio (HL motion ratio—hyoid bone displacement divided by laryngeal displacement) as an index of swallowing. The HL motion ratio is independent of physiological changes associated with age and height and shows an index of 0.50 for normal swallowing [19]. Deviations from a HL motion ratio of 0.50 may be associated with dysphagia; however, it currently remains unknown how much such deviation causes dysphagia. NOD is a motion dysfunction in the oropharynx during swallowing and, thus, affects the displacement of both the hyoid bone and laryngeal. In the present study, we compared and analyzed the displacement and duration of the hyoid bone and larynx during swallowing as well as the HL motion ratio in a healthy group and dysphagia group.

## Materials and methods

### Participants

Thirty-six participants were examined between October 2018 and April 2020: 18 healthy male elderly participants (76.5±7.6 years old, the healthy group) and 18 male stroke patients diagnosed with NOD (76.3±11.6 years old, the dysphagia group). The exclusion criteria of the healthy group included common influential factors, such as a history of head and neck surgery, malignant tumors, respiratory disease, and swallowing adjustments required in daily diets. The exclusion criteria of the dysphagia group included a history of neuromuscular disease, which is generally considered to affect dysphagia, an inability to sit in a wheelchair for more than 30 minutes, low stability in the head and neck, severe dysphagia with an inability to perform oral ingestion, the need for nasal administration, and poor cognitive function of those who cannot follow inspection instructions. All participants in the dysphagia group were diagnosed with NOD using the VF test conducted by a doctor and speech therapist. The severity of dysphagia was based on either occasional or water aspiration in the Dysphagia Outcome and Severity Scale [21]. Healthy participants, patients, and their families were informed purpose of the present study and provided with an advance consent form. The present study was approved by the Kansai University of Welfare Sciences Ethics committee (18–24). Written informed consent was obtained from all participants for the publication of this study and accompanying images.

### Evaluation of swallowing function

Participants swallowed 5 mL of liquid and water with an adjusted viscosity and the movements of the hyoid bone and larynx were visualized by US. The healthy group was asked to swallow liquid, while the dysphagia group swallowed water with an adjusted viscosity based on their individual ability. This was performed three to five times. Three of these image sets, in which the movements of the hyoid bone and larynx were sufficiently tracked, were selected for analysis.

The participant assumed a posture with the neck in a neutral position such that a line from the acromion to the opening of the ear canal was at 100°–110° to the horizontal plane. The head and neck were fixed with a base to minimize movement. A 5- to 12-MHz linear probe (Digital Color Doppler Ultrasound System JS2; SonoScape Medical Corp, Centennial, CO, USA) was attached to the left or right plate of the thyroid cartilage without disturbing the laryngeal elevation while swallowing. In order to visualize larynx motions while swallowing, US images are adjusted to show the anterior angle of the thyroid cartilage. The third right of the monitor showed the upper thyroid cartilage and the overall image is adjusted to display the hyoid bone and larynx. The hyoid bone was identified as a high echo area with a rear acoustic shadow on US. The measurement point was the anterior inferior margin of the hyoid bone. The left of the monitor showed the cranial portion, while the right showed the caudal portion. The thyroid cartilage was identified as a low echo area and the measurement point was the tip of the thyroid cartilage.

### Image analysis and parameter measurements

**Fig 1:**
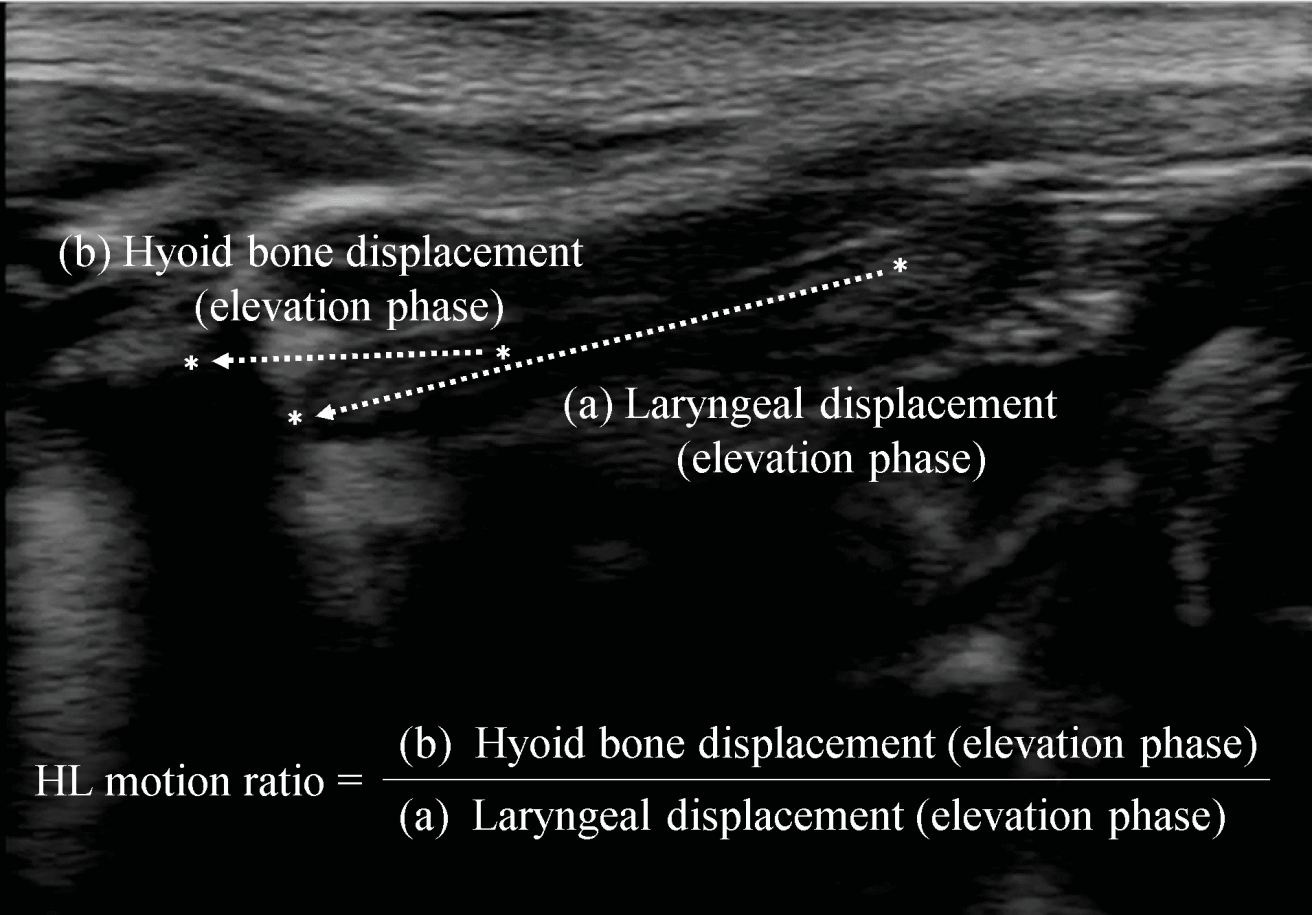
Visualization of the hyoid bone and larynx by ultrasonography, and the HL motion ratio. (A) Distance from the resting position at which elevation of the upper end of the thyroid cartilage reaches the maximally elevated position, at which elevation stops.(B) Distance from the resting position at which elevation of the hyoid bone reaches the maximally elevated position, at which elevation stops.

Acquired images were converted into an audio video interleave format and transferred from the hard drive of the US device to a personal computer, on which they were analyzed using two-dimensional data analysis software (Dipp Motion Ver 1.1.31; DITECT Co., Tokyo, Japan). Markers were set at the anterior inferior margin of the hyoid bone and uppermost end of the larynx, and measurement points were automatically tracked in each frame using the tracking function of the software. Vertical and anteroposterior directions were considered to be the x- and y-axes, respectively, and the distances moved in these directions were measured. When hyoid bone movement was accompanied by an instantaneous shadow, this was manually corrected. Swallowing movement was measured from the initiation of the laryngeal elevation to the completion of the downward laryngeal movement. The elevation and descending phases were the respective periods from the start point to the maximally elevated position then back to the static position. The displacement of the hyoid bone and larynx was measured in the elevation and descending phases. The HL motion ratio was calculated by dividing hyoid bone displacement (elevation phase) by laryngeal displacement (elevation phase) as the swallowing index (Fig 1). The parameters examined in the present study were the displacement of the hyoid bone and larynx during swallowing and their durations (including the “static phase”, which is the static time at the maximally elevated position of the hyoid bone and larynx) as well as the HL motion ratio. In addition, basic information on participants such as height, weight, and age were recorded and displayed as an average ± SD.

### Statistical analysis

Basic information on the healthy and dysphagia groups, the displacement and duration of the hyoid bone and larynx, and the HL motion ratio were compared using a mann—whitney *U* test. Significant items were used as independent variables and the rate of dysphagia as a dependent variable in the multiple regression analysis to investigate their effects on the evaluation of dysphagia. Significant items in the dysphagia evaluation were placed in a Receiver Operation Characteristic Curve (ROC curve) for illuminate the area under the curve (AUC), cut-off value, sensitivity, and specificity. Statistical analyses were performed using IBM SPSS Statistics for Windows, Version 24.0 (IBM Corp., Armonk, NY, USA), and the significance level was set at p < 0.01.

## Results

Table 1 shows the basic information of the two groups, the displacement and duration of the hyoid bone and larynx during swallowing, and the HL motion ratio. No significant differences were observed in age, height, or weight between the groups, whereas significant differences were noted in laryngeal duration (static phase), laryngeal displacement (elevation phase), and the HL motion ratio. Although laryngeal duration (static phase) was longer in the dysphagia group than in the healthy group, laryngeal displacement (elevation phase) was greater in the healthy group than in the dysphagia group. Laryngeal displacement (elevation phase) in the healthy group is longer than in the dysphagia group. However, there were no significant differences in the HL motion ratio of the hyoid bone displacement (elevation phase) between the healthy and disabled groups.

**Table 1:** Results of Mann—Whitney *U* test between the healthy and dysphagia groups.

		Healthy group (*n* = 18)	Dysphagia group (*n* = 18)	*P*-value
Characteristics	Age (years)	76.5 ± 7.6	76.3 ± 11.6	0.791
	Weight (kg)	56.1 ± 6.8	56.2 ± 9.1	0.888
	Height (cm)	159.4 ± 9.8	162.0 ± 4.9	0.584
Laryngeal duration	Elevation phase (sec)	0.50 ± 0.12	0.68 ± 0.32	0.055
	Static phase (sec)	0.19 ± 0.06	0.29 ± 0.12	0.012*
	Descending phase (sec)	0.64 ± 0.34	0.67 ± 0.26	0.606
Laryngeal displacement	Elevation phase (mm)	20.9 ± 4.1	14.7 ± 4.7	0.000**
	Descending phase (mm)	17.9 ± 4.2	14.9 ± 5.1	0.085
Hyoid bone duration	Elevation phase (sec)	0.47 ± 0.14	0.59 ± 0.43	0.743
	Static phase (sec)	0.26 ± 0.12	0.33 ± 0.12	0.091
	Descending phase (sec)	0.56 ± 0.21	0.63 ± 0.33	0.424
Hyoid bone displacement	Elevation phase (mm)	10.5 ± 2.3	11.0 ± 3.9	0.606
	Descending phase (mm)	8.2 ± 2.3	10.3 ± 4.1	0.091
HL motion ratio		0.51 ± 0.07	0.76 ± 0.19	< 0.000**

* *P* < .05,

** *P* < .001.

Therefore, the HL exercise ratio calculated by applying laryngeal displacement (elevation phase) to the denominator was larger in the dysphagia group than in the healthy group. Laryngeal displacement (elevation phase) in the healthy group is longer than in the dysphagia group. However, there were no significant differences in the HL motion ratio of the hyoid bone displacement (elevation phase) between the healthy group and the disabled group. Therefore, the HL exercise ratio calculated by applying laryngeal displacement (elevation phase) to the denominator was larger in the dysphagia group than in the healthy group. The distance of the laryngeal elevation was shorter in the dysphagia group than in the healthy group, with no significant differences in the time from the laryngeal static phase to the maximally elevated position.

Table 1 shows basic information on the two groups, the displacement and duration of the hyoid bone and larynx during swallowing, and the HL motion ratio.

Table 2 shows the results of the multiple regression analysis with dysphagia as the dependent variable. There were significant differences between the groups in laryngeal duration (static phase), laryngeal displacement (elevation phase), and HL motion ratio. Thus, in the multivariate analysis, these three variables were defined as independent variables and dysphagia occurrence was defined as a dependent variable. The analysis revealed that there was a significant association (F = 16.2, p<.001, R2 = .603). Laryngeal displacement (elevation phase) significantly differed between the healthy and dysphagia groups, but did not affect the evaluation of dysphagia. Therefore, laryngeal duration (static phase) and the HL motion ratio were associated with the presence or absence of NOD.

**Table 2:** Results of the multiple regression analysis with dysphagia as the dependent variable.

	Standard regression coefficient	Standard error	t value	P-value
Section		.412	-.405	.688
Laryngeal duration (Static phase)	.295	.539	2.569	.015*
Laryngeal displacement (Elevation phase)	−.267	.125	-2.014	.053
HL motion ratio	.471	.345	3.574	< 0.001**
R-squared value	.603			

* *P* < .05,

** *P* < .001.

**Fig 2:**
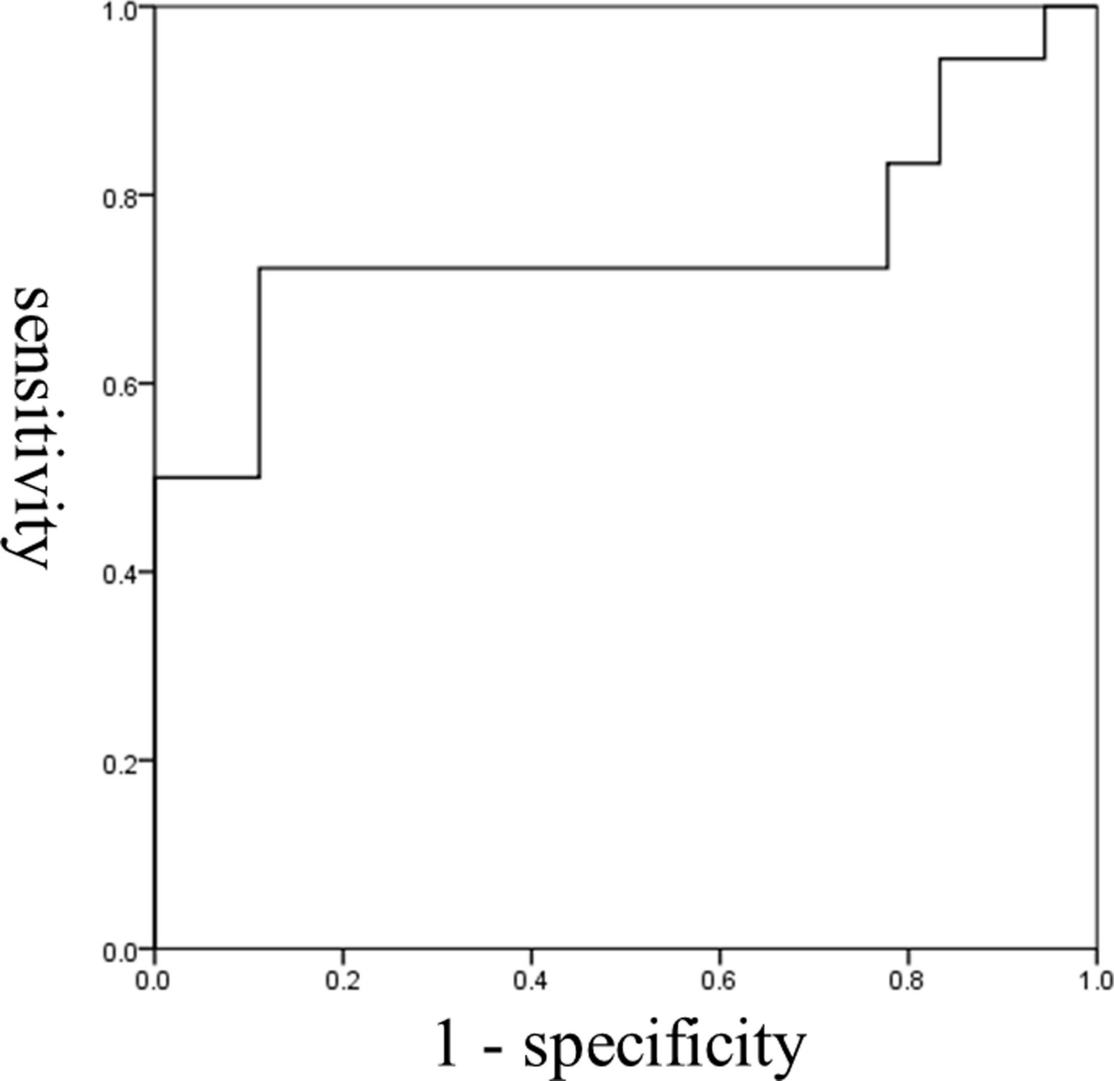
ROC curve for laryngeal duration (static phase). The cut-off value was 0.26 sec (sensitivity, 72.2%; specificity, 88.9%). The area under the ROC curve was 0.744; Standard error 0.091; Significance 0.012; Confidence interval 0.566–0.922.

**Fig 3:**
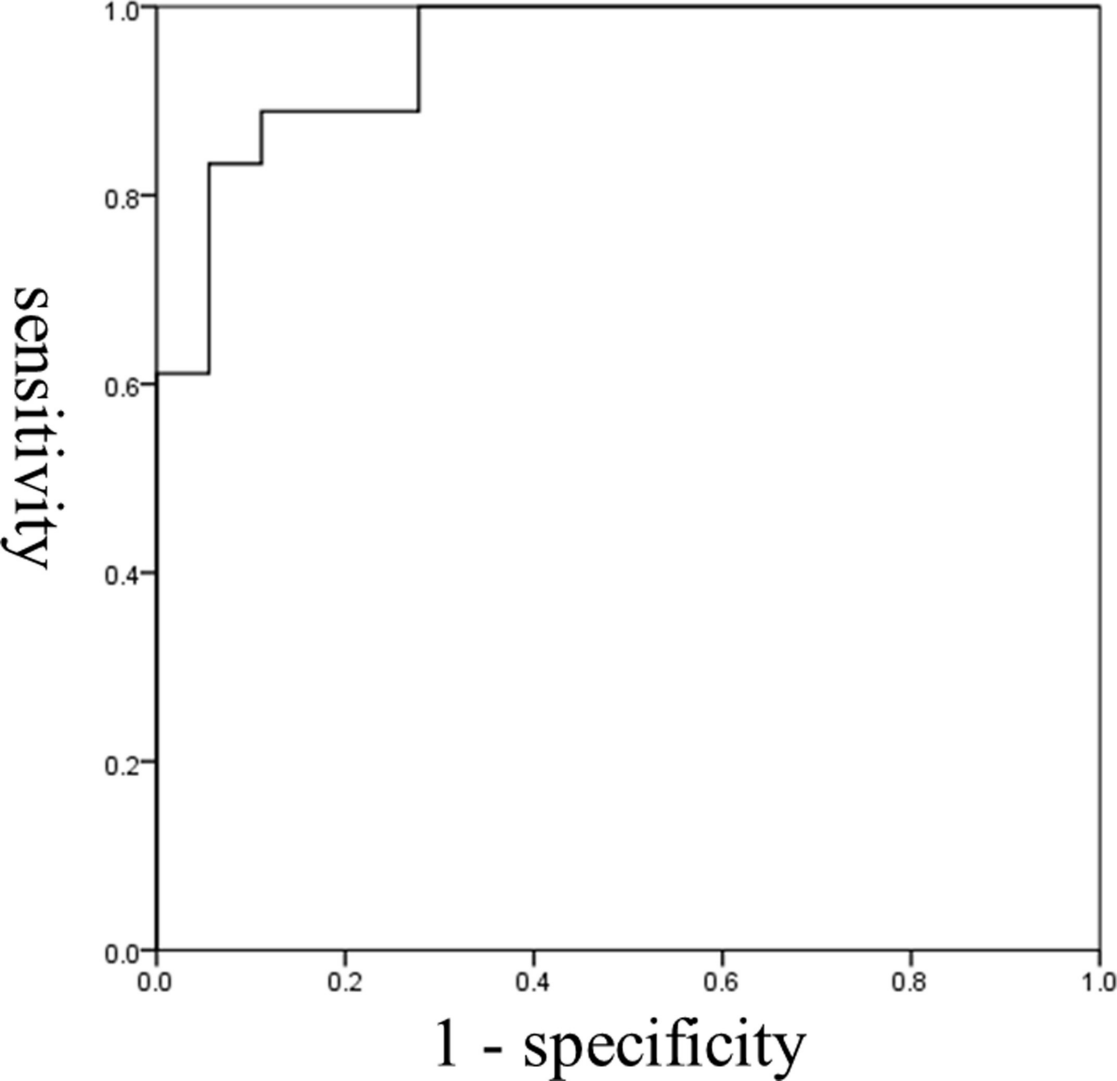
ROC curve for the HL motion ratio. The cut-off value was 0.56 (sensitivity, 88.9%; specificity, 88.9%). The area under the ROC curve was 0.951; Standard error 0.032; Significance 0.000; Confidence interval 0.887–1.000.

The ROC curves of the two variables that affect the detection of dysphagia are shown in Figs 2 and 3. AUC for laryngeal duration (static phase) was 0.744 and the cut-off value based on the Youden Index was 0.26 sec. Sensitivity based on this cut-off was 72.2% and specificity was 88.9%. Regarding the HL motion ratio, AUC was 0.951, the cut-off value based on the Youden Index was 0.56. Sensitivity based on this value was 88.9% and specificity was 88.9%.

## Discussion

The present study investigated the ability of participants to swallow 5 mL of liquid. The healthy group swallowed liquid and the dysphagia group swallowed water with an adjusted viscosity based on their individual ability. Regarding the effects of viscosity on hyoid bone and laryngeal displacement, Lee et al. [22] revealed in a VF test that viscosity did not affect the motion or elevation distance of the larynx. Steele et al. [23] also demonstrated that viscosity did not affect the maximally elevated position of the hyoid bone. Since the viscosity of concentrated and diluted liquids did not significantly alter the larynx volume contraction rate [24], we concluded that displacement of the hyoid bone and larynx was not affected by viscosity. The dysphagia group in this study was examined using the VF test to assess the severity of dysphagia. The viscosity was adjusted so that all participants could safely swallow 5 mL of water.

### Evaluation of swallowing movement by US

During the swallowing process, the hyoid bone initially moves upward, then forward, and returns down to its original position [25,26]. Okada et al. [27] reported that the forward motion of the hyoid bone and thyrohyoid muscle contraction occurred simultaneously in a muscle length analysis using 320-row area detector computed tomography. In the present study, the measurement starting point was set to the initiation of the laryngeal elevation, and hyoid bone displacement during the elevation phase was considered to correspond to the forward movement of the hyoid bone. The upward movement of the hyoid bone is movements of the digastric and mylohyoid muscles, while the forward movement of the hyoid bone is achieved by the geniohyoid muscle [27]. Since hyoid bone displacement in the present study was the distance of the forward motion of the hyoid bone, the HL motion ratio reflected cooperation between the thyrohyoid muscle, which elevates the larynx, and the geniohyoid muscle, which moves the hyoid bone forward. In our previous study in which healthy participants were asked to swallow 5 mL liquid, the HL motion ratio was 0.50 [19].

Laryngeal displacement is an important index among the many indications of pulmonary aspiration. When the larynx is elevated, the anterior larynx closes, which opens the entrance of the esophagus. It is a key structural element for protecting the respiratory tract against aspiration [28]. Symptoms of NOD can be related to swallowing dysfunction, such as delayed anterior larynx closure and weak tongue pressure, as well as reduced larynx sensation and motor disorder of the central nervous system [29]. A delay in anterior larynx closure and larynx elevation during swallowing results in a longer bolus duration in the larynx, which increases the risk of aspiration [30]. In the present study, laryngeal displacement (elevation phase) was shorter and the larynx elevation speed was slower in the dysphagia group than in the healthy group. Laryngeal displacement (elevation phase) in the dysphagia group is consistent with the features of NOD. Nevertheless, the results of the multiple regression analysis adjusted for confounding factors indicated that laryngeal displacement (elevation phase) was not related to the occurrence of dysphagia. Furthermore, no significant difference was observed in hyoid bone displacement between the healthy and dysphagia groups. A compensatory strategy to elevate the hyoid bone to a higher position is needed to ensure that the upper part of the esophagus remains open in healthy elderly participants in whom the failure of this strategy may lead to dysphagia [31]. In the present study, adjustments to the viscosity of water based on the severity of dysphagia represented a successful compensatory strategy for liquid intake, such that the same extent of hyoid bone displacement as the healthy group was maintained.

Based on the present results, the single evaluation of hyoid bone or laryngeal displacement does not accurately evaluate swallowing function. The success of oropharyngeal swallowing is associated with the motion of the hyoid bone and larynx [32,33]; therefore, any incompatibility may increase the risk of pulmonary aspiration. An index that reflects cooperation between the hyoid bone and larynx during swallowing is needed. Picelli et al. [34] reported that the results of conventional swallowing screening tests were associated with hyoid-larynx approximation when the larynx is maximally elevated during swallowing. Similar to the present study, they also used this association as an indicator of coordinated movement between hyoid bone and larynx.

When the cut-off value of the HL motion ratio in the present study was 0.56 or higher, it predicted dysphagia. Laryngeal duration (static phase) is less effective than the HL motion ratio, but may also be used to predict dysphagia. Hyoid bone displacement and its elevation speed did not significantly differ between the healthy and dysphagia groups, while laryngeal displacement (elevation phase) was shorter in the dysphagia group than in the healthy group, and the laryngeal elevation speed was also slower. Compared with healthy individuals, patients with post-stroke dysphagia have reduced duration of glottal closure and opening of the esophagus, as well as shorter larynx elevation distance [35]. Participants in the dysphagia group in the present study swallowed their adjusted daily diets through oral ingestion and safety was ensured by adjustments to liquid viscosity. The dysphagia group had shorter laryngeal displacement and a slower elevation speed than the healthy group, while hyoid bone displacement was similar. Therefore, the extension of the static phase when the larynx is at its maximally elevated position may be a compensatory strategy for the insufficient elevation of the larynx when a bolus passes through. When the laryngeal duration (static phase) was 0.26 sec or longer, it predicted dysphagia. It is important to note that the risk of aspiration increases when the larynx compensatory strategy does not function well. The extension of the laryngeal duration (static phase) appeared to be a feature of dysphagia in the present study, but it does not guarantee the detection of dysphagia. This is also the case for the HL motion ratio in which changes may be due to the severity of dysphagia.

The water drinking test is currently widely used in the clinical field for swallowing screening [36]. This test may also be employed to diagnose dysphagia based on aspiration clinical signs, such as suffocation, coughs, and a wet voice [37]. Assessments also include differential diagnosis from dysphagia caused by organic causes such as pharyngeal tumors [38–40]. Since US provides an objective assessment of the motion of the hyoid bone and larynx and may help to detect NOD, it may be added to the current swallowing screening test for the more detailed management [41,42] of dysphagic patients.

### Limitations of this study and future prospects

The present study has a number of limitations. While the severity of poststroke dysphagia varies, this study adopted a dysphagia group with the same severity, which was biased towards severe cases. Furthermore, the number of participants was insufficient to cover the wide range of severities; therefore, the number of cases needs to be increased in order to improve screening accuracy. Moreover, screening accuracy was based on diagnosed cases of dysphagia only, and, thus, needs to be performed to detect dysphagia in the clinical field for further verification.

## Supporting information

S1 File (XLSX)
